# Does Citrulline Have Protective Effects on Liver Injury in Septic Rats?

**DOI:** 10.1155/2016/1469590

**Published:** 2016-04-19

**Authors:** Bin Cai, Yu-long Luo, Shi-jun Wang, Wei-yuan Wei, Xue-hui Zhang, Wei Huang, Tong Li, Meng Zhang, Nan Wu, Gopaul Roodrajeetsing, Sen Zhang

**Affiliations:** ^1^Department of Colorectal Surgery, First Affiliated Hospital of Guangxi Medical University, Nanning, Guangxi 530021, China; ^2^Department of Gastrointestinal Surgery, First Affiliated Hospital of Guangxi Medical University, Nanning, Guangxi 530021, China; ^3^Department of Nuclear Medicine, People's Hospital of Beihai City, Beihai, Guangxi 536000, China; ^4^Department of Neurosurgery, First Affiliated Hospital of Guangxi Medical University, Nanning, Guangxi 530021, China

## Abstract

Citrulline (Cit) supplementation was proposed to serve as a therapeutic intervention to restore arginine (Arg) concentrations and improve related functions in sepsis. This study explored whether citrulline had positive effects on liver injury and cytokine release in the early stages of sepsis. The cecal ligation and puncture (CLP) model was utilized in our study. Rats were divided into four groups: normal, Cit, CLP, and CLP+Cit. The CLP group and CLP+Cit group were separated into 6-, 12-, and 24-hour groups, according to the time points of sacrifice after surgery. Intragastric administration of L-citrulline was applied to rats in Cit and CLP+Cit groups before surgery. Serum AST and ALT levels and levels of MDA, SOD, NO, and iNOS in the liver tissues were evaluated. Plasma concentrations of Cit and Arg were assessed using HPLC-MS/MS. Serum concentrations of cytokines and chemokines were calculated by Luminex. Results showed SOD activities of CLP+Cit groups were significantly higher than that of CLP groups, contrasting with the MDA and NO levels which were significantly lower in CLP+Cit groups than in CLP groups. In addition, plasma concentrations of TNF-*α*, IL-6, and IL-1*β* were significantly lower in the CLP+Cit 6-hour group than in the CLP 6-hour group.

## 1. Introduction

Prospective studies investigating arginine (Arg) metabolism revealed that plasma arginine concentrations decreased significantly in sepsis patients [1, 2]. A meta-analysis containing 341 patients demonstrated that, compared with control group, plasma concentrations of arginine in sepsis patients decreased by 41% [3]. Therefore, some researchers put forward that arginine supplementation could be a logical therapeutic option to restore the decreased arginine concentrations in sepsis [4]. However, clinical randomized controlled trials showed arginine supplementation had no significant improvement on hospital mortality, infectious complications, or the length of ICU stays in sepsis patients [5]. Another study reported that, for patients with severe sepsis, arginine supplementation led to a significantly higher mortality rate [6]. This may be due to arginine supplementation having generated a lot of nitric oxide (NO) in the role of inducible nitric oxide synthase (iNOS), leading to a decrease in blood pressure and an increased burden on heart blood vessels, eventually resulting in a lack of oxygen supply in tissues and organs [7]. L-arginine can serve as a substrate for iNOS, generating NO and L-citrulline. However, the structures of L-citrulline and L-arginine are very similar. If the L-citrulline cannot be cleared in time, the accumulation of L-citrulline may competitively inhibit the enzyme activity center. Furthermore, L-citrulline can be recycled to L-arginine during reactions catalyzed by argininosuccinate synthetase (ASS) and argininosuccinate lyase (ASL). All of the above constitute the so-called “Cit-NO cycle” [8]. Therefore, citrulline supplementation was proposed to serve as a therapeutic intervention to restore arginine concentrations in sepsis.

Sepsis-associated liver injury is one of the most common complications in sepsis. It has been reported that the incidence of sepsis-associated liver injury was 34.7%, and sepsis-associated liver injury is a significant predictive sign of poor prognosis in sepsis patients [9]. One piece of research from France including 206 ICUs showed that in the first 24 hours of ICU stay the incidence of liver damage and liver failure was 46.6% and 6.3%, respectively [10]. The liver is the major organ of substance and energy metabolism, as well as the major source of cytokines. During the early stages of sepsis, the body is at a proinflammatory stage accompanied by the release of a large number of proinflammatory cytokines such as TNF-*α*, IL-1*β*, and IL-6. These proinflammatory cytokines could activate a cytokine cascade reaction and lead to “cytokine storm,” aggravating inflammatory and immune response [11]. In our previous research, we provided L-citrulline to cecal ligation and puncture- (CLP-) induced septic rats and found that citrulline supplementation reduced the reaction of proinflammatory mediators [12]. In the present study, through supplying L-citrulline to CLP rats, we tried to explore whether citrulline supplementation had positive effects on liver injury and the release of cytokines in the early stages of sepsis.

## 2. Materials and Methods

### 2.1. Animals

120 male Wistar rats, 7 to 10 weeks old and weighing 250~300 g, were provided by the Experimental Animal Center of Guangxi Medical University. All rats were housed in animal care facilities and maintained under pathogen-free conditions. Rats were reared in strict accordance with standard operating procedures. Ambient temperature was kept at 22~25°C, humidity at 45~55%, with a 12-hour light/12-hour dark light cycle. Timely water addition and feeding were managed daily and litter was cleaned once every two days. Rats were euthanized by an intraperitoneal injection of chloral hydrate (700 mg/kg) if they appeared to show agonal breathing or a body weight loss greater than 20%. Animals' health was specifically monitored and recorded once every six hours.

### 2.2. Study Design

According to a random number table, rats were randomly divided into following groups: normal group (*n* = 8), Cit group (*n* = 8), CLP group (*n* = 52), and CLP+Cit group (*n* = 52). CLP group and CLP+Cit group were then separated into 6-, 12-, and 24-hour groups, according to the time points of sacrifice after CLP operation, that is, CLP 6 h group (*n* = 12), CLP 12 h group (*n* = 16), CLP 24 h group (*n* = 24), CLP+Cit 6 h group (*n* = 12), CLP+Cit 12 h group (*n* = 16), and CLP+Cit 24 h group (*n* = 24). Intragastric administration of L-citrulline (purity ≥ 99%, RUIBIO, Germany) was supplied to the rats in Cit group and CLP+Cit groups for 7 days before further operation (a dose of 200 mg kg^−1^·d^−1^).

### 2.3. CLP Model

The procedure of CLP modeling was performed as previously described [12]. Rats were weighed and anesthetized by intraperitoneal injection with 10% chloral hydrate at a dose of 0.35 mL/100 g body weight. After shaving and disinfecting the abdomen, a 1.5~2 cm incision on the anterior abdominal wall was performed for exposing and extracting the cecum colon. After gently pushing cecum contents to the distal end of the cecum, the distal 1/3 of the cecum was ligated with a 4-0 silk suture and punctured through one time using a 12-gauge needle. A small amount of intestinal contents was gently squeezed from the punctured holes. After suturing the incision, sterile saline solution was offered to rats subcutaneously for resuscitation at a dose of 4 mL/100 g body weight. For the sham group, in addition to the cecal ligation and puncture, the remaining operations were the same as CLP modeling groups. The general condition and survival status of rats were observed once every 6 hours after surgery.

### 2.4. Sample Collection

For CLP groups and CLP+Cit groups, six surviving rats were randomly selected and sacrificed 6, 12, and 24 hours after surgery. After anesthesia, blood was collected via the abdominal aorta and liver tissues were removed. Tissues were immediately frozen at −80°C for further analysis. Blood was used to extract serum and plasma. Serum levels of aspartate aminotransferase (AST) and alanine aminotransferase (ALT) were measured using an automatic biochemical analyzer (Beckman DXC800, USA). Plasma concentrations of arginine and citrulline were assessed by the method of HPLC-MS/MS.

### 2.5. MDA, SOD, NO, and iNOS Levels Evaluation

The enzymatic activities of superoxide dismutase (SOD) and the levels of malondialdehyde (MDA) in the liver tissues were evaluated by a microplate reader (SpectraMax Plus 384 MD, USA) using commercial reagent kits from Nanjing Jiancheng Bioengineering Institute. NO levels and iNOS activities in the liver tissues were measured by NO colorimetric assay kit and NOS Assay Kit, respectively (Nanjing Jiancheng Bioengineering Institute, China).

### 2.6. HPLC-MS/MS Assay

Plasma concentrations of arginine and citrulline were assessed by the method of HPLC-MS/MS. All experimental operations were done by China National Analytical Center (Guangzhou, China). HPLC-MS/MS analysis was performed using an Agilent® 1200LC/6410B HPLC system (Agilent Technologies Inc., Wilmington, USA). The analytical column was Agilent Poroshell 120 HILIC (2.1 mm × 100 mm, 2.7 *μ*m). The column temperature was room temperature. The chromatographic run was carried out at 0.3 mL/min as the flow rate, with 0.2% aqueous formic acid as mobile phase A and acetonitrile as mobile phase B. The injection volume was 3 *μ*L. The ion source was electrospray ionization ion source (ESI) in the positive mode. The drying gas (N_2_) temperature was 350°C, atomization gas (N_2_) pressure was 40 psi, drying gas (N_2_) flow was 10.0 L/min, and the electrospray ionization voltage was 4000 V. Scan mode was multiple reactions monitoring (MRM) mode.

### 2.7. Serum Cytokines and Chemokines Concentrations Measurement

Serum concentrations of cytokines and chemokines were measured by Milliplex MAP Rat Cytokine/Chemokine Magnetic Bead Panel (RECYMAG65K27PMX, Millipore, USA) following the kit-specific protocols provided by Millipore. The kit was used for the simultaneous quantification of the following cytokines and chemokines: EGF, Eotaxin, Fractalkine, G-CSF, GM-CSF, GRO/KC/CINC-1, IFN-*γ*, IL-1*α*, IL-1*β*, IL-2, IL-4, IL-5, IL-6, IL-10, IL-12(p70), IL-13, IL-17A, IL-18, IP-10, Leptin, LIX, MCP-1, MIP-1*α*, MIP-2, RANTES, TNF-*α*, and VEGF. 96-well plates were read using Luminex FLEXMAP 3D*™* (Luminex, Austin) with xPONENT 4.2 software (Luminex, Austin). Data was analyzed using Milliplex Analyst software (Millipore, USA). A five-parameter logistic regression model with weighting was utilized to create standard curves. Two quality controls were executed with each assay.

### 2.8. Statistical Analysis

All values were showed as mean ± standard deviation (SD). Data was analyzed by chi-square test, Student's *t*-test, and one-way ANOVA. All data was statistically analyzed using SPSS 16.0 software (SPSS, Chicago, USA). *P* < 0.05 was regarded as statistically significant.

## 3. Results

### 3.1. General Performances and Intra-Abdominal Lesions of Rats

Six~twelve hours after surgery, all rats from the CLP groups and CLP+Cit groups appeared apathetic and unresponsive, showed reduced activity, towering hair, prone and shrinking body, and sticky secretions around the nose and mouth, and were drinking less. Rats displayed drowsiness, indifferent responses to external stimuli, perianal purulent discharge, and hemorrhaging eyes 24 hours after surgery. Intraperitoneal bloody ascites was observed in the CLP model rats, accompanied with stench, intestinal inflation, and cecal ligation segment necrosis. The liver presented edema, congestion, and visible point flaky bleeding in the CLP model rats. The above symptoms were not observed in the normal and Cit groups. In addition, liver morphology was also normal with no pathological changes in the normal and Cit groups.

### 3.2. Serum Levels of AST and ALT

Table 1 shows the serum levels of ALT and AST in each group. There were no significant differences in serum levels of ALT and AST between the normal and Cit groups (*P* > 0.05). ALT and AST levels of CLP and CLP+Cit groups were much higher than that of the normal group. In addition, at 6, 12, and 24 hours after surgery, there was no significant difference in serum ALT levels between CLP groups and CLP+Cit groups (all *P* > 0.05). However, serum AST levels in CLP+Cit 12- and 24-hour groups were significantly lower than that in CLP 12- and 24-hour groups (both *P* < 0.05).

### 3.3. SOD Activities and MDA Levels in the Liver Tissues

Table 2 shows the SOD activities and MDA levels in the liver tissues of each group. There were no significant differences in SOD activities and MDA levels between the normal group and Cit group (*P* > 0.05). At 6, 12, and 24 hours after surgery, SOD activities of CLP+Cit groups were significantly greater than that of CLP groups (all *P* < 0.05). In contrast, MDA levels in CLP+Cit 12- and 24-hour groups were significantly lower than that in CLP 12- and 24-hour groups (both *P* < 0.05). Citrulline supplementation remarkably reduced the production of MDA and improved the SOD activities in septic rats.

### 3.4. NO Levels and iNOS Activities in the Liver Tissues

Table 3 shows the NO levels and iNOS activities in the liver tissues of each group. The normal and Cit groups showed no significant differences in NO levels and iNOS activities (*P* > 0.05). At 6, 12, and 24 hours after surgery, NO levels in CLP+Cit groups were significantly lower than that in CLP groups (all *P* < 0.05). However, at the three time points, there was no significant difference in iNOS activities between CLP groups and CLP+Cit groups (all *P* > 0.05).

### 3.5. Plasma Concentrations of Cit and Arg

**Figure 1:**
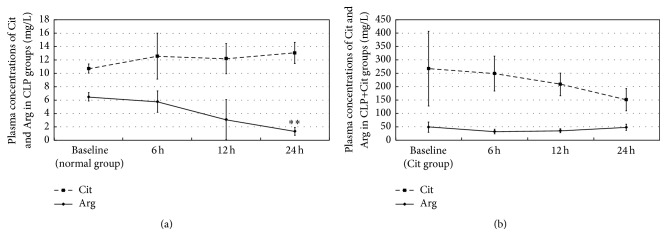
Plasma concentrations of Cit and Arg in CLP groups and CLP+Cit groups. (a) Plasma Arg concentration of CLP 24-hour group was significantly lower than the baseline (normal group) (^*∗∗*^ *P* < 0.01). (b) Plasma Arg concentration of CLP+Cit 24-hour group remained near the baseline (Cit group).

As shown in Figure 1(a), after CLP surgery, plasma concentration of Arg declined in CLP groups. At 24 hours after surgery, plasma Arg concentration of CLP 24-hour group was significantly decreased compared with the baseline (*P* < 0.01). Interestingly, in the first 24 hours after surgery, when compared to the baseline plasma Cit concentrations increased slightly in CLP groups, but without statistical significance. In our study, citrulline supplementation remarkably increased plasma concentrations of Arg and Cit in CLP+Cit groups. In contrast to CLP groups, Cit concentrations declined in CLP+Cit groups. In addition, plasma Arg concentration of CLP+Cit 24-hour group remained near the baseline 24 hours after surgery. Our results suggested that citrulline supplementation alleviated the lack of arginine in CLP-induced septic rats.

### 3.6. Serum Cytokines and Chemokines Concentrations

**Figure 2:**
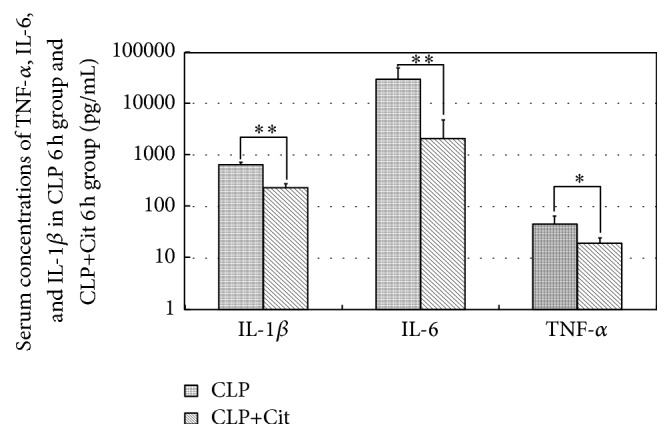
Serum concentrations of TNF-*α*, IL-6, and IL-1*β* in CLP+Cit 6-hour and CLP 6-hour groups. At 6 hours after surgery, concentrations of TNF-*α*, IL-6, and IL-1*β* were significantly lower in CLP+Cit 6-hour group than CLP 6-hour group; ^*∗*^ *P* < 0.05, ^*∗∗*^ *P* < 0.01.

Table 4 shows the serum concentrations of cytokines and chemokines in each group. Serum concentrations of all cytokines and chemokines were higher in the CLP 6-hour group than that in the normal group, wherein 21 kinds of cytokines and chemokines increased significantly (*P* < 0.05), including EGF, Eotaxin, G-CSF, GM-CSF, GRO/KC/CINC, IFN-*γ*, IL-1*α*, IL-1*β*, IL-2, IL-4, IL-5, IL-6, IL-10, IL-17A, IP-10, Leptin, MCP-1, MIP-1*α*, MIP-2, RANTES, and TNF-*α*, suggesting that our modeling was satisfactory. Using mean values as references, in the first 24 hours after surgery, concentrations of 20 kinds of cytokines and chemokines (EGF, Eotaxin, G-CSF, IL-1*β*, IL-2, IL-4, IL-5, IL-6, IL-10, IL-12(p70), IFN-*γ*, IL-17, Leptin, MCP-1, MIP-1*α*, MIP-2, TNF-*α*, VEGF, IL-13, and Fractalkine) peaked earlier in the CLP group compared with CLP+Cit group. Only two cytokines (GM-CSF and IP-10) peaked earlier in CLP+Cit group. The remaining five kinds of cytokines and chemokines (IL-1*α*, GRO/KC/CINC-1, LIX, RANTES, and IL-18) reached peak at the same time point in CLP group and CLP+Cit group. This may be due to citrulline supplementation postponing the releases of cytokines and chemokines in the early stages of sepsis. In addition, it was noteworthy that concentrations of proinflammatory cytokines TNF-*α*, IL-6, and IL-1*β* were significantly lower in the CLP+Cit 6-hour group than in the CLP 6-hour group (all *P* < 0.05). Citrulline supplementation reduced the release of TNF-*α*, IL-6, and IL-1*β* in the early stages of sepsis (Figure 2).

## 4. Discussion

Sepsis is a complex syndrome with a variety of clinical manifestations. The cecal ligation and puncture method used in our study has been recognized as the “gold standard” in sepsis research throughout the past three decades [13]. Inflammation can induce changes in the amino acid profile, which is pronounced in sepsis. A previous study measured the changes in the plasma amino acid concentrations of sepsis patients and found that citrulline, glutamate, and arginine showed the greatest decreases when compared with concentrations in healthy control subjects. The decreased degrees of citrulline, glutamate, and arginine were 56%, 48%, and 47%, respectively [2]. Arginine is a nonessential amino acid which can be derived from daily dietary intake,* de novo* synthesis from citrulline, and protein breakdown [4]. Arginine is involved in protein synthesis and secretion of NO, creatine, urea, and amines. In addition, arginine plays an important role in cell regeneration, wound healing, cellular immune function, protein conversion, and the maintenance of positive nitrogen balance [14]. In sepsis, a decline in arginine intake, accompanied with an impaired arginine* de novo* synthesis and reduced protein intake and absorption, causes endogenous arginine production to only 30% of normal levels [15]. In addition, lipopolysaccharide can promote the release of iNOS and accelerate the consumption of arginine in the development of sepsis [16].

There has been no doubt that citrulline supplementation could enhance plasma and tissue arginine concentrations. At the beginning of the century, citrulline as a precursor amino acid of arginine had been given orally in a clinical trial, resulting in elevated plasma levels of arginine [17]. A subsequent study further demonstrated that oral citrulline supplementation raised plasma arginine concentrations in a dose-dependent manner [18]. In our previous study, we found that serum arginine concentrations were greater in the citrulline-supplemented rats compared to rats on a standard “rat chow” diet [12]. Therefore, citrulline supplementation has been seen as an effective method to improve arginine concentrations. In our study, plasma arginine concentrations decreased in CLP groups but remained at the baseline in CLP+Cit groups, suggesting that citrulline supplementation alleviated the lack of arginine in CLP-induced septic rats. Interestingly, citrulline concentrations in CLP groups slightly increased in the first 24 hours after surgery, but without statistical significances. This may be due to the monitoring period of our study, which was only 24 hours long.

The liver is one of the most vulnerable organs in sepsis. The mechanism of liver injury in sepsis is complex, involving hepatic microcirculation disorders, releases of inflammatory mediators, activation of Kupffer cells, induced apoptosis of liver cells, and so forth. ALT and AST are the most commonly used indicators of hepatocellular damage. The distributions of ALT and AST in the liver cells are different. ALT are mainly distributed in the cytoplasm of hepatocytes; however, AST are mainly distributed in the mitochondria. In our study, ALT and AST levels of CLP and CLP+Cit groups were significantly higher than that of the normal group, indicating that hepatocytes were damaged in CLP modeling rats. There were no statistical differences in serum ALT levels between CLP and CLP+Cit groups, suggesting that the degree of damage to the integrity of hepatocytes in both groups was about the same. However, serum AST levels of CLP+Cit groups were significantly lower than that of CLP groups, indicating that the degree of damage to the integrity of mitochondria in CLP+Cit groups was much lighter than that in CLP groups. Citrulline supplementation reduced mitochondrial damage in septic rats. SOD is one of the common antioxidants in hepatocytes; our results showed SOD activities in CLP+Cit groups were significantly higher than that in CLP groups, suggesting that citrulline supplementation enhanced SOD activities in sepsis. MDA is one of the cell membrane lipid peroxidation end-products, which can reflect the degree of membrane lipid peroxidation and the damage extent of cell membranes. In our study, MDA levels in CLP+Cit groups were significantly lower than that in CLP groups, suggesting that citrulline supplementation reduced the cell membrane lipid peroxidation.

The liver has a large number of Kupffer cells, which account for about 80% of the total fixed macrophage population [19]. Bacteria and endotoxins can activate Kupffer cells, inducing excessive releases of oxygen radicals, NO, and inflammatory cytokines such as IL-1, IL-6, and TNF-*α* [20]. Oxygen radicals can attack cell membranes and induce lipid peroxidation, generate a large number of metabolites, and cause damage to the integrity of membranes, leading to cell and mitochondrial swelling and resulting in liver damage eventually [21]. NO is a freely diffusible small molecule. The half-life of NO is only a few seconds, but NO can react with superoxide anion fast, forming peroxynitrite which can cause damage to cells [22]. The mass production of NO in sepsis can lead to hepatic microcirculation disorders. NO can combine with mitochondrial cytochrome oxidase and inhibit its activity, blocking the electron transfer chain and resulting in the formation of superoxide [23]. Mitochondrial damage can induce the accumulation of neutrophils in the liver, forming a vicious cycle. In addition, NO can reduce the production rate of glycogen in hepatocytes and result in hypoglycemia in sepsis [24]. Our results showed NO levels of CLP+Cit groups were significantly lower than that of CLP groups, suggesting that citrulline supplementation reduced the production of NO in septic rats. In our study, there was no statistically significant difference in iNOS activities between CLP+Cit groups and CLP groups, but lower iNOS activities were detected in CLP+Cit groups compared with CLP groups. The iNOS activity values in CLP 6-, 12-, and 24-hour groups were 0.42 ± 0.11, 0.41 ± 0.18, and 0.43 ± 0.18, respectively. However, values in CLP+Cit 6-, 12-, and 24-hour groups were 0.34 ± 0.08, 0.33 ± 0.11, and 0.38 ± 0.06, respectively. This may partly explain why less NO was produced in CLP+Cit groups.

Proinflammatory cytokines TNF-*α*, IL-1*β*, and IL-6 are produced in the initial phase of sepsis. They can stimulate the innate or adaptive immune response, characterized by further production of many kinds of cytokines, exacerbating the inflammatory response [25]. In addition, TNF-*α* and IL-1*β* can enhance the expressions of adhesion molecules such as intercellular adhesion molecule-1 (ICAM-1) and vascular cell adhesion molecule-1 (VCAM-1), aggravating hepatic microcirculation disorders [26]. IL-1*β* and TNF-*α* can also increase integrin adhesiveness in neutrophils and promote their extravasation into tissues, causing organ damage [27]. Moreover, the combination of TNF-*α* and TNF receptor-1 can induce apoptosis of hepatocytes through multiple apoptotic pathways [28]. In our study, concentrations of TNF-*α*, IL-6, and IL-1*β* were significantly lower in the CLP+Cit 6-hour group than in the CLP 6-hour group. This suggested that citrulline supplementation reduced the release of TNF-*α*, IL-6, and IL-1*β* in the early stages of sepsis. In addition, Luminex results showed concentrations of 20 kinds of cytokines and chemokines reached peak earlier in CLP groups compared to CLP+Cit groups, whereas only two cytokines peaked earlier in CLP+Cit groups. We estimated that citrulline supplementation postponed the release of cytokines and chemokines in the early stages of sepsis.

## 5. Conclusions

In conclusion, our study revealed that citrulline supplementation reduced the damage to mitochondria in hepatocytes. In addition, citrulline supplementation reduced the cell membrane lipid peroxidation and enhanced the SOD activities in septic rats. In the early stages of sepsis, the production of NO was decreased and the release of TNF-*α*, IL-6, and IL-1*β* was inhibited by citrulline supplementation.

## Figures and Tables

**Table 1:** Serum levels of ALT and AST in each group.

Group	ALT (U/L)			AST (U/L)		
	6 h	12 h	24 h	6 h	12 h	24 h
Normal	56 ± 8.32			244.17 ± 123.95		
Cit	59 ± 9.53			316.67 ± 54.14		
CLP	120.5 ± 41.37	169.67 ± 27.83	278.5 ± 84.28	433.00 ± 73.72	631.83 ± 111.96	1231.17 ± 220.86
CLP+Cit	102.67 ± 17.04	158.17 ± 43.72	240 ± 64.15	370.83 ± 167.36	456 ± 129.71^*∗*^	788.83 ± 123.78^*∗∗*^

Comparisons between CLP groups and CLP+Cit groups at the same time point; ^*∗*^ *P* < 0.05, ^*∗∗*^ *P* < 0.01.

**Table 2:** SOD activities and MDA levels in the liver tissues.

Group	SOD (U/mgprot)			MDA (nmol/mgprot)		
	6 h	12 h	24 h	6 h	12 h	24 h
Normal	344.27 ± 26.42			1.09 ± 0.14		
Cit	358.05 ± 18.42			1.05 ± 0.17		
CLP	285.97 ± 65.10	303.93 ± 13.86	260.67 ± 60.01	1.55 ± 0.17	1.77 ± 0.11	1.97 ± 0.14
CLP+Cit	402.90 ± 30.08^*∗∗*^	358.48 ± 35.83^*∗*^	392.38 ± 37.06^*∗∗*^	1.37 ± 0.26	1.42 ± 0.18^*∗∗*^	1.53 ± 0.29^*∗∗*^

Comparisons between CLP groups and CLP+Cit groups at the same time point; ^*∗*^ *P* < 0.05, ^*∗∗*^ *P* < 0.01.

**Table 3:** NO levels and iNOS activities in the liver tissues.

Group	NO (nmol/mgprot)			iNOS (U/mgprot)		
	6 h	12 h	24 h	6 h	12 h	24 h
Normal	1.53 ± 0.46			0.13 ± 0.04		
Cit	1.63 ± 0.46			0.14 ± 0.04		
CLP	3.90 ± 1.18	5.04 ± 1.99	5.55 ± 1.55	0.42 ± 0.11	0.41 ± 0.18	0.43 ± 0.18
CLP+Cit	1.93 ± 0.82^*∗*^	2.73 ± 1.53^*∗∗*^	3.41 ± 0.94^*∗∗*^	0.34 ± 0.08	0.33 ± 0.11	0.38 ± 0.06

Comparisons between CLP groups and CLP+Cit groups at the same time point; ^*∗*^ *P* < 0.05, ^*∗∗*^ *P* < 0.01.

**Table 4:** Serum concentrations of cytokines and chemokines in each group (pg/mL).

Analyte	Normal group	Cit group	CLP group			CLP+Cit group		
			6 h	12 h	24 h	6 h	12 h	24 h
G-CSF	Undetected	Undetected	8.75 ± 2.38	13.59 ± 2.01^▲^	13.08 ± 0.08	4.72 ± 3.92	4.03 ± 1.62	21.38 ± 0.71^▲^
Eotaxin	6.65 ± 2.83	3.78 ± 1.57	14.89 ± 4.78^▲^	12.24 ± 7.88	10.49 ± 2.99	12.22 ± 2.59	12.46 ± 5.30	17.35 ± 4.95^▲^
GM-CSF	Undetected	Undetected	98.58 ± 92.66	105.45 ± 86.76^▲^	72.70 ± 22.15	82.70 ± 53.41^▲^	63.39 ± 36.31	49.49 ± 23.77
IL-1*α*	3.10 ± 2.19	Undetected	55.67 ± 26.83^▲^	23.86 ± 9.70	41.49 ± 24.61	53.31 ± 48.32^▲^	26.85 ± 22.37	51.55 ± 21.02
Leptin	2810.2 ± 1184.97	2123.75 ± 699.71	20809.5 ± 7712.10^▲^	15190.6 ± 7086.61	6007.75 ± 4217.84	8735.2 ± 1996.72	9794 ± 5442.86	18706.6 ± 13241.32^▲^
MIP-1*α*	32.67 ± 11.55	26.36 ± 3.51	513.14 ± 287.45^▲^	127.97 ± 87.43	36.86 ± 12.72	205.86 ± 88.74	183.71 ± 193.66	251.80 ± 196.25^▲^
IL-4	Undetected	Undetected	29.49 ± 22.55^▲^	19.71 ± 20.95	8.18 ± 12.72	24.60 ± 11.00	31.65 ± 21.72	39.54 ± 19.73^▲^
IL-1*β*	49.28 ± 42.19	44.16 ± 14.61	652.95 ± 54.49^▲^	371.35 ± 289.02	301.4 ± 234.89	233.07 ± 45.24	152.92 ± 117.18	278.02 ± 185.33^▲^
IL-2	37.92 ± 17.30	25.92 ± 6.67	170.84 ± 75.04^▲^	71.37 ± 68.65	65.11 ± 25.37	63.95 ± 33.33	94.15 ± 2.50^▲^	92.90 ± 63.45
IL-6	120.95 ± 25.71	64.18 ± 43.70	30070 ± 20440.72^▲^	1954.90 ± 923.72	1293.21 ± 934.89	2097.75 ± 2727.34	1688.46 ± 1826.3	21660.26 ± 27160.17^▲^
EGF	0.71 ± 0.177	Undetected	2.22 ± 0.35^▲^	1.13 ± 1.88	1.59 ± 1.33	0.11 ± 0.08	0.85 ± 1.21	1.40 ± 1.33^▲^
IL-13	19.14 ± 3.90	10.29 ± 5.82	22.72 ± 12.89	22.83 ± 9.28^▲^	15.98 ± 3.83	24.38 ± 4.70	20.37 ± 9.06	31.31 ± 13.92^▲^
L-10	53.06 ± 23.80	62.83 ± 28.28	2254.67 ± 1025.29^▲^	2067.44 ± 2493.23	502.68 ± 208.96	321.36 ± 65.49	725.92 ± 613.66	2459.21 ± 1949.54^▲^
IL-12(p70)	77.31 ± 22.39	77.80 ± 0.76	193.73 ± 124.92^▲^	148.91 ± 88.35	58.43 ± 64.20	111.88 ± 34.80	108.50 ± 47.57	168.61 ± 56.16^▲^
IFN-*γ*	56.042 ± 23.74	43.32 ± 15.73	125.1 ± 52.58^▲^	102.26 ± 42.89	85.74 ± 37.04	87.24 ± 39.86	98.87 ± 52.01	120.82 ± 57.06^▲^
IL-5	58.71 ± 21.54	50.30 ± 18.33	127.96 ± 30.39	132.35 ± 28.57^▲^	107.46 ± 10.24	105.54 ± 16.63	122.15 ± 26.10	140.97 ± 18.23^▲^
IL-17A	8.35 ± 3.91	3.70 ± 2.09	36.44 ± 30.49^▲^	29.68 ± 20.33	14.53 ± 4.73	19.65 ± 6.78	15.37 ± 3.42	30.52 ± 13.88^▲^
IL-18	136.57 ± 42.88	157.14 ± 85.53	471.45 ± 354.16	436.92 ± 374.40	640.50 ± 669.11^▲^	453.14 ± 383.18	214.26 ± 197.21	459.29 ± 305.39^▲^
MCP-1	855.72 ± 309.68	750.93 ± 219.38	7939.5 ± 5748.21	8296.6 ± 7103.81^▲^	5456.4 ± 2145.50	5685.4 ± 2610.89	7344.4 ± 2128.33	7420 ± 3687.26^▲^
IP-10	291.16 ± 76.83	241.01 ± 62.06	921.03 ± 458.42	979.95 ± 352.73^▲^	601.56 ± 241.94	867.48 ± 143.13^▲^	653.79 ± 180.21	822.54 ± 274.09
GRO/KC	12.23 ± 8.76	17.15 ± 6.99	16361 ± 14266.49^▲^	5538.8 ± 5664.49	3875 ± 3330.03	6199.4 ± 4313.97^▲^	4605.4 ± 2235.93	3894.75 ± 3154.25
VEGF	52.39 ± 21.31	52.0 ± 8.25	139.35 ± 111.78^▲^	97.94 ± 71.38	111.59 ± 93.36	94.86 ± 21.80	63.03 ± 21.77	106.72 ± 57.30^▲^
Fractalkine	80.86 ± 31.82	60.21 ± 10.99	131.05 ± 80.13	134.01 ± 135.06^▲^	73.80 ± 31.21	118.60 ± 40.96	69.76 ± 19.78	128.01 ± 75.52^▲^
LIX	2671 ± 172.81	2458.5 ± 347.71	3629.25 ± 859.08^▲^	3116.4 ± 983.28	3111.4 ± 1181.79	3815.6 ± 1137.01^▲^	3286.4 ± 796.47	3307.8 ± 796.00
MIP-2	91.92 ± 31.31	65.76 ± 16.40	5966 ± 3067.43^▲^	621.04 ± 280.54	371.54 ± 380.89	1844.39 ± 1099.42	840.10 ± 547.80	3875.67 ± 2332.24^▲^
TNF-*α*	8.76 ± 1.17	5.35 ± 3.98	46.40 ± 20.48^▲^	40.76 ± 48.68	13.26 ± 1.40	20.02 ± 5.13	25.33 ± 17.72	49.52 ± 38.76^▲^
RANTES	2235.8 ± 385.35	2161 ± 537.21	3981.25 ± 779.13^▲^	2488.4 ± 1003.48	2629.4 ± 876.18	3949 ± 1156.80^▲^	3259.2 ± 1015.13	2318.6 ± 1027.21

Serum concentrations of cytokines and chemokines were undetected if less than the minimum detectable concentration; ▲ represented the concentration peaks of cytokines and chemokines within 24 hours after surgery in CLP group and CLP+Cit group.
